# Tuning the Coordination Environment of Ru(II) Complexes with a Tailored Acridine Ligand

**DOI:** 10.3390/molecules29153468

**Published:** 2024-07-24

**Authors:** Ali Awada, Pierre-Henri Lanoë, Christian Philouze, Frédérique Loiseau, Damien Jouvenot

**Affiliations:** Université Grenoble Alpes, CNRS, DCM, 38000 Grenoble, France; ali.awada.chem@gmail.com (A.A.); pierre-henri.lanoe@univ-grenoble-alpes.fr (P.-H.L.); christian.philouze@univ-grenoble-alpes.fr (C.P.)

**Keywords:** tridentate ligands, coordination geometry, ruthenium(II), acridine-based ligand, NMR spectroscopy, X-ray analyses

## Abstract

A novel tridentate ligand featuring an acridine core and pyrazole rings, namely 2,7- di-*tert-*butyl-4,5-di(pyrazol-1-yl)acridine, **L**, was designed and used to create two ruthenium(II) complexes: [Ru**L**_2_](PF_6_)_2_ and [Ru(tpy)**L**](PF_6_)_2_. Surprisingly, the ligand adopted different coordination modes in the complexes: facial coordination for the homoleptic complex and meridional coordination for the heteroleptic complex. The electronic absorption and electrochemical properties were evaluated. Although both complexes exhibited favorable electronic properties for luminescence, neither emitted light at room temperature nor at 77 K. This study highlights the complex interplay between ligand design, coordination mode, and luminescence in ruthenium(II) complexes.

## 1. Introduction

A fascinating aspect of coordination chemistry is how the properties of a complex can be drastically modified by subtle changes on the ligands. Ligands can be meticulously crafted in order to achieve desired functions when associated with the appropriate metal. In the realm of photochemistry, ruthenium(II) stands out and has been combined with a wide range of ligands. Most photoactive complexes are based on a tris(diimine) octahedral structure, such as the well-known [Ru(bpy)_3_]^2+^ (bpy = 2,2′-bipyridine) [1]. Even if the synthetic availability of tailored bidentate ligands has improved [2], the use of tridentate ligands would provide more stability, symmetry, and linearity to the corresponding complexes. Nevertheless, bis(tridentate) Ru^II^ complexes display poor photophysical properties, as proven by the weak emitting properties of [Ru(tpy)_2_]^2+^ (tpy = 2,2′;6′,2″-terpyridine) at room temperature. This loss of emission is attributed to the distortion caused by the tridentate ligand, leading to a weaker ligand field and thus allowing for easy population of the ^3^MC (metal-centered) state from the ^3^MLCT (metal-to-ligand charge transfer) state via internal conversion [3].

To overcome this limitation, Hammarström’s team proposed a strategy in 2006 involving six-membered chelate rings [4], which provide an optimal geometry for hosting the Ru^II^ ion in a near-perfect octahedral environment, limiting the ^3^MC state’s population. Since then, this approach has been widely adopted to enhance the photophysical properties of Ru(II) complexes [5,6]. However, achieving long emission lifetimes and high quantum yields requires careful consideration of the electronic properties of the ligand. Specifically, the energy of the ^3^MC state relative to the emissive ^3^MLCT state needs to be high to prevent internal conversion. Simply raising both energy levels simultaneously does not improve the outcome [7]. Therefore, the electronic properties of the ligand, particularly the presence of low-lying π* orbitals, are crucial. Ligands with large aromatic surfaces [8], such as those based on the acridine motif, are advantageous in this regard but have been underutilized in complex design [9,10,11]. Our group proposed a tridentate ligand incorporating an acridine core flanked by two pyridine rings [12]. However, the strain and torsion of the acridine platform upset its electronic properties.

This study introduces a new ligand where the pyridine rings have been replaced with smaller pyrazole heterocycles. This geometric change would offer a more suitable coordination nest to the Ru^II^ ion. Both homo- and heteroleptic complexes have been prepared and characterized. This work is part of Dr. Ali Awada’s PhD thesis [13].

## 2. Results and Discussion

### 2.1. Syntheses

**Scheme 1:**
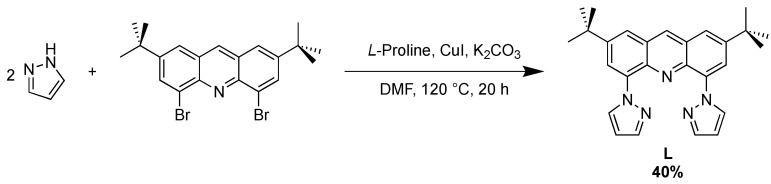
Synthesis of ligand **L**.

The synthesis of the novel target ligand, 2,7- di-*tert-*butyl-4,5-di(pyrazol-1-yl)acridine, **L**, as illustrated in Scheme 1, was conducted straightforwardly. A single synthetic step sufficed to produce the new ligand from the previously prepared intermediate. The di-brominated compound underwent Ullman-type amination with a 1*H*-pyrazole heterocycle in the presence of copper(I) iodide and anhydrous potassium carbonate in dry dimethylformamide at 120 °C under an argon atmosphere overnight. Established protocols were adapted to afford ligand **L** as a pale yellow solid in a 40% yield [14].

**Scheme 2:**
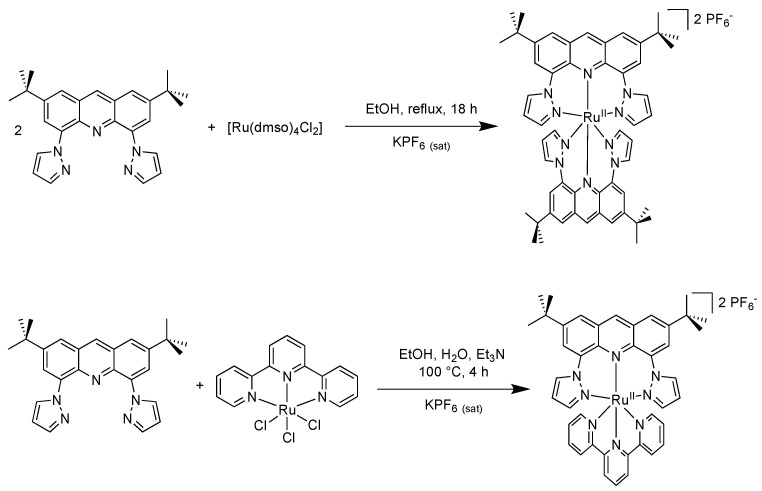
Syntheses of complexes [Ru**L**_2_](PF_6_)_2_ and [Ru(tpy)**L**](PF_6_)_2_.

The homoleptic complex [Ru(**L**)_2_]^2+^ was synthesized by the reaction of **L** with *cis-*[RuCl_2_(dmso)_4_] in a molar ratio of 2:1 in refluxing EtOH overnight as illustrated in Scheme 2. On cooling, the solution deposited a blue precipitate of this complex, which was subsequently purified by chromatography. Following anion metathesis, the complex was isolated with PF_6_^−^ as counterions in a 35% yield.

In parallel, the heteroleptic analog possessing the co-ligand terpyridine was produced from the reaction between [RuCl_3_(tpy)] and **L** in equimolar amounts using Et_3_N as a reducing agent. The reaction mixture was heated under reflux in an EtOH/H_2_O mixture (70/30, *v*/*v*). After purification and anion exchange, the complex [Ru(tpy)**L**](PF_6_)_2_ was obtained as a blue solid in a 29% yield.

### 2.2. Characterization

**Figure 1:**
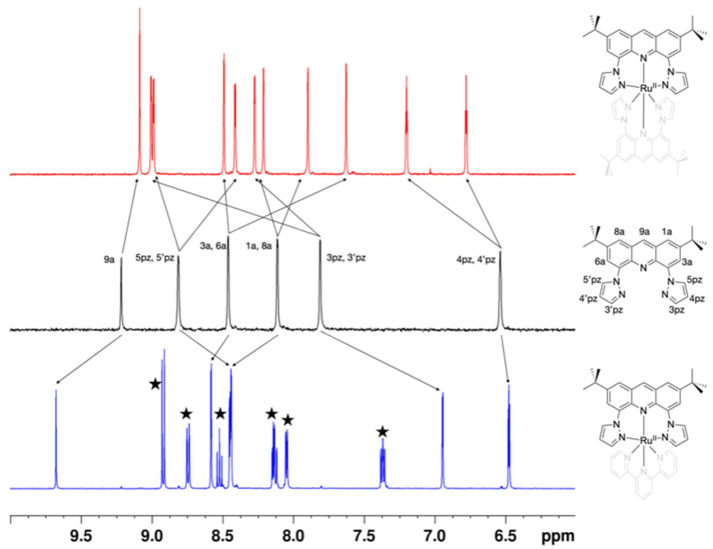
The assigned aromatic region of the ^1^H-NMR spectra of the complex [Ru(**L**)_2_]^2+^ (red top), ligand **L** (black middle), and complex [Ru(tpy)**L**]^2+^ (blue bottom) recorded in acetone-*d*_6_ at 500 MHz. Signals with an asterisk indicate peaks attributable to the tpy ligand.

In all ^1^H-NMR spectra, the relative integration of the peaks featured in the aromatic region aligns with the integration of the easily recognizable *t*Bu signal (an intense peak in the high-field region). A striking feature of the ^1^H-NMR spectrum of the homoleptic complex resides in the clear splitting of the ligands’ signals into two sets of peaks. The uncoordinated ligand **L** features the classical set of signals including six peaks in the aromatic region in addition to the characteristic 18 isochronous -*t*Bu protons. Except for proton 9a, all other peaks appear as narrow doublets. This is consistent with the *J*^4^ coupling for the protons of the acridine heterocycle. For the pyrazole ring, it has been reported that *J^3^* coupling constant values lie between 1 and 2 Hz [15]. The expected meridional geometry of our initial design would have provided a similar set of peaks only featuring a mere shift stemming from the coordination to the metal center. A *fac-anti* isomer, featuring S_4_ symmetry, would have displayed a simple spectrum as well. However, direct evidence of the C2-*fac* geometry was discerned from the ^1^H-NMR spectrum, as depicted in Figure 1, wherein all proton signals in the spectrum of the symmetrical ligand **L** (red top) are separated into two sets of peaks (except for the central acridine proton) in the spectrum of the homoleptic complex [Ru(**L**)_2_]^2+^ (black middle). In addition, NOESY experiments were performed (Figure S3), revealing a strong correlation between the *t*Bu protons and proton 9 from the acridine heterocycle, as well as protons 4,4′ from the pyrazole rings. Such interactions are not conceivable inside one single ligand. They stem necessarily from the interaction between two inequivalent ligands on the complex.

**Figure 2:**
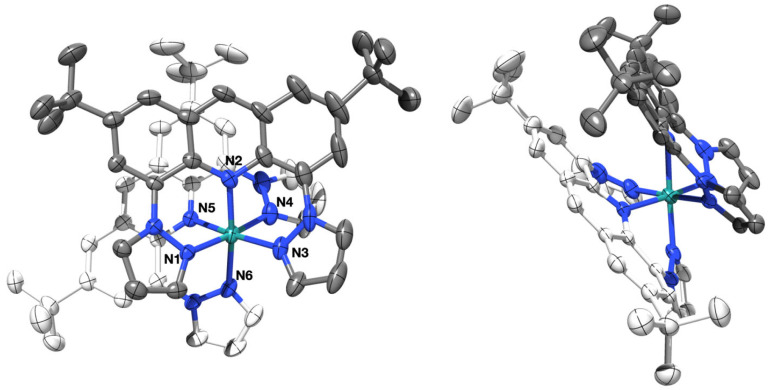
Different projections of the crystal structure of the complex [Ru(**L**)_2_]^2+^ (40% ellipsoids). Solvent molecules (co-crystallized acetone molecules), hydrogen atoms, and PF_6_^−^ counterions are omitted for clarity.

X-ray diffraction of a single crystal, grown by slowly evaporating an acetone solution of the complex, confirmed the proposed geometry (shown in Figure 2 with labeled coordinating atoms). A black plate-shaped crystal of 0.16 × 0.36 × 0.58 mm was selected for X-ray data collection at 200 K. It showed an orthorhombic crystal system of space group *Pbca* and an asymmetric unit featuring C_54_H_58_N_10_Ru displaying significant disorder from C10 to C27 including N3 and N8 atoms, two (PF_6_^−^) counterions, of which only one of them is not disordered, and one co-crystallized acetone molecule (C_3_H_6_O). The crystal packing relies in part on π-π stacking based on the acridine platform. The structure reveals that both pyrazole rings from the same ligand and both acridine units are arranged in a *cis* fashion around the central Ru^II^ ion. The bond lengths are presented in Table 1 and are comparable with those of similar complexes previously described featuring a facial coordination mode [16]. The angles between these Ru-bonded atoms are approximately 94° and 93° for the pyrazoles and acridines, respectively. This finding confirms the unsymmetrical facial (*fac*) geometry of the complex.

Despite the surprising geometry, the coordination sphere around the Ru^II^ ion is not very distant from ideal measurements (see Table 1). Therefore, photophysical studies of this blue complex were carried out and are discussed later in this manuscript.

To achieve the meridional coordination of ligand **L**, a heteroleptic complex was designed with a terpyridine as the second ligand. Indeed, the terpyridine ligand occupies three positions on the metal in a meridional way, leaving only one possibility for the coordination of a second tridentate ligand. The ^1^H-NMR spectrum of [Ru(tpy)**L**]^2+^ confirmed the *mer* coordination. In this case, contrary to the splitting observed for the homoleptic complex, ligand **L** shows C2 symmetry. Compared to the free ligand (Figure 1), coordination to the [Ru(tpy)] fragment induces mainly downfield shifts, with the particular exception of protons 3,3′ of the pyrazole. After coordination, these protons now lie in the shielding cone induced by the facing terpyridine.

**Figure 3:**
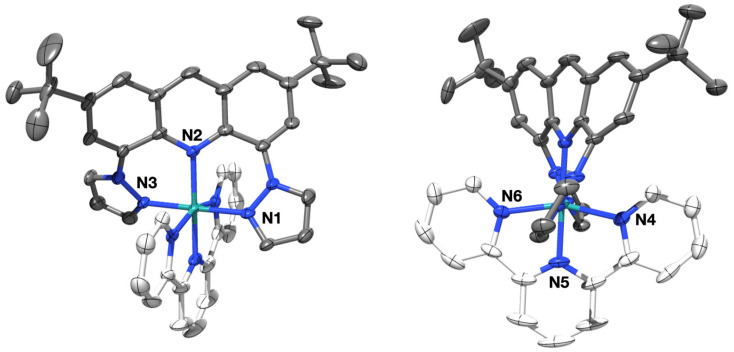
Different projections of the crystal structure of the complex [Ru(tpy)**L**]^2+^ (40% ellipsoids). Solvent molecules (co-crystallized acetone molecules), hydrogen atoms, and PF_6_^−^ counter anions are omitted for clarity.

X-ray diffraction analysis of a single crystal grown by exposing a solution of [Ru(tpy)**L**](PF_6_)_2_ in acetonitrile to diethyl ether vapors confirmed the complex’s structure (Figure 3). A violet needle-shaped crystal of 0.12 × 0.22 × 0.50 mm was chosen for X-ray data collection at 200 K. It displayed a monoclinic crystal system of space group *C2*/*c* and an asymmetric unit consisting of C_42_H_40_N_8_Ru featuring a disordered *tert-*butyl group at C21, two disordered PF_6_^−^ counterions, and one co-crystallized acetone molecule (C_3_H_6_O) spread over three main positions. The coordination angles and bond lengths are presented in Table 1. In this case, the twisting observed on the acridine heterocycle prevents efficient π-π stacking. Interestingly, the molecular structure reveals two distinct coordination environments around the ruthenium center. One side, where ligand **L** binds, exhibits near-perfect octahedral geometry with angles close to 90° and 180° and bond lengths comparable to those of previously described related structures [12]. The other side, bonded to the terpyridine (tpy), shows the typical distortions often observed with such ligands [17]. Indeed, the angles on this side of the molecules display values far from the ideal geometry (around 80° and 160°). It is interesting and somewhat surprising to observe that despite its apparent rigidity, ligand **L** is able to coordinate to the metal center in two different modes.

### 2.3. UV-Vis Spectroscopy

**Figure 4:**
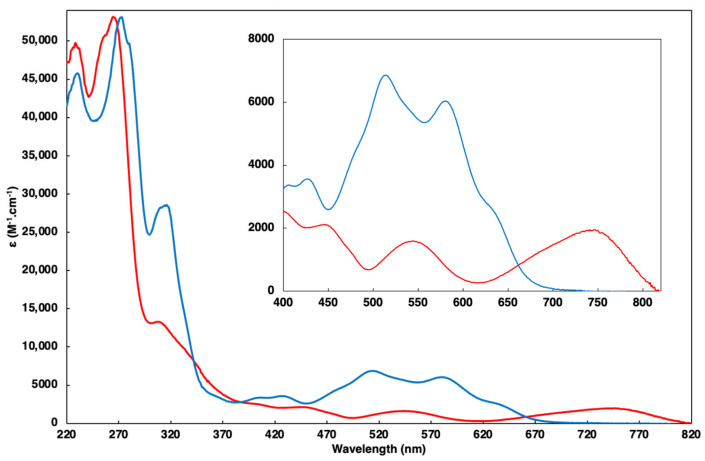
The UV–visible absorption spectra of [Ru(**L**)_2_]^2+^ (red) and [Ru(tpy)**L**]^2+^ (blue) recorded in an acetonitrile solution. Inset: zoom on the visible region of the spectra.

The photophysical characteristics of both complexes were studied thoroughly. The UV–visible absorption spectra recorded in the acetonitrile solution are depicted in Figure 4 and the data are gathered in Table 2. As for classical polyimine complexes, the spectra feature intense bands in the UV region generally assigned to spin-allowed ligand-centered transitions.

The metal-to-ligand charge transfer (MLCT) transitions, observed in the visible region, are pivotal as they are at the origin of the emission properties of ruthenium complexes. These transitions, denoted as M(d)-L(π*), are notably red-shifted in both complexes relative to the reference [Ru(tpy)_2_]^2+^ complex (λ_abs_ = 475 nm) [18]. This red shift is rationalized by the smaller HOMO-LUMO energy gap in both complexes compared to the reference complex [1]. This finding is consistent with the electrochemical data that show a reversible oxidation peak attributed to the Ru^III^/Ru^II^ couple at a lower potential for each compound. These values can be construed by the fact that for ligand **L**, the better alignment of the coordinating atoms allows for a better overlap and thus a more donating character. Logically, the complex carrying two ligands **L** undergoes a larger effect than the heteroleptic complex. Reduction processes involving ligand **L** are irreversible and occur at less negative potentials compared to the tpy ligand. Table 3 summarizes the electrochemical data obtained by cyclic voltammetry (see the CV traces in the Supplementary Materials), and Table 4 compares the HOMO-LUMO energy gaps estimated from UV-Vis and electrochemical data.

The moderately intense MLCT absorption of the complex [Ru(**L**)_2_]^2+^ spans from 500 nm to 820 nm, with maxima at 540 nm attributed to Ru-to-pyrazole ring transition and 747 nm attributed to Ru-to-lowest acridine π* orbitals transitions. On the other hand, the complex [Ru(tpy)**L**]^2+^ displays a lesser red-shifted MLCT band compared to [Ru(tpy)_2_]^2+^, ranging from 400 nm to 750 nm, with maxima at 514 nm and 580 nm. The higher-energy transition (514 nm) corresponds to the metal-to-terpyridine ligand transition, while the second (580 nm) is attributed to the metal-to-lower π* orbitals transitions localized on ligand **L**. These higher energies can be construed by the globally fewer donations of the ligand set associated with a distorted acridine moiety displaying lower aromaticity and therefore higher-energy π* orbitals.

The excitation of both [Ru(**L**)_2_]^2+^ and [Ru(tpy)**L**]^2+^ complexes at their respective ^1^MLCT wavelengths in the visible region did not yield any emission, neither at room temperature in acetonitrile nor at 77 K in a butyronitrile rigid matrix. This behavior could be assigned to numerous factors. For the homoleptic complex, the low energy of the HOMO-LUMO transition, stemming from an adequate coordination geometry combined with a planar large aromatic ligand, suggests that the luminescence is quenched in virtue of the energy gap law. In the case of [Ru(tpy)**L**]^2+^, the loss of luminescence could in part be assigned to non-radiative deactivation pathways induced by the twisting of the acridine. Additionally, a direct intersystem crossing from the ^1^MLCT to the non-emitting lowest-lying triplet state of the acridine heterocycle cannot be ruled out given the relative energies [19].

## 3. Materials and Methods

### 3.1. Materials

All chemicals were purchased from Sigma-Aldrich (Burlington, MA, USA) and used as received. 4,5-Dibromo-2,7-di-*tert*-butylacridine was synthetized according to procedures in the literature [12].

### 3.2. Physicochemical Measurements

^1^H NMR and ^13^C NMR spectra were recorded on Bruker 400 or 500 MHz spectrometers (Billerica, MA, USA). ^1^H and ^13^C chemical shifts (ppm) were referenced to residual solvent peaks [20]. The electrospray ionization mass spectrometry (ESI-MS) analysis was performed on an Amazon Speed (Bruker Daltonics)–Ion Trap Spectrometer. Absorption spectra were recorded on a Varian Cary 300 Scan UV–visible spectrophotometer (Palo Alto, CA, USA). Emission spectra were recorded on a Horiba Scientific FluoroMax-4 spectrofluorimeter (Kyoto, Japan). Samples in acetonitrile solutions were placed in 1 cm path length quartz cuvettes for room temperature measurements, and in a butyronitrile rigid matrix for 77 K measurements. Electrochemical measurements were recorded using a CHI-620B potentiostat (CH Instruments, Bee Cave, TX, USA). The concentration was 10^−3^ M, with a sweep rate = 100 mV×s^−1^. Tetra-*n*-butylammonium hexafluorophosphate was used as the supporting electrolyte (0.1 M) in dry CH_3_CN. A standard three-electrode electrochemical cell was used, potentials were referenced to a Ag/AgNO_3_ reference electrode, and the working electrode was a 3 mm diameter Pt disk electrode (E_pa_: anodic peak potential; *E*_pc_: cathodic peak potential; *E*_1/2_ = (*E*_pa_ + *E*_pc_)/2; Δ*E*_p_ = *E*_pa_ − *E*_pc_). Experimental uncertainties are as follows: absorption maxima, ±2 nm; redox potentials, ±10 mV.

### 3.3. X-ray Structure Determinations

Crystals of [Ru(**L**)_2_](PF_6_)_2_ and [Ru(tpy)**L**](PF_6_)_2_ were selected, damped in a paraffin mixture, mounted on a nylon cryo-loop, and then centered at 200 K on a Bruker-AXS-enraf-nonius Kappa goniometer equipped with a high-brilliance micro-source with Mo Kα radiation (λ = 0.71073 Å). The data were collected with an APEXII 2D detector, then integrated and corrected for Lorentz and polarization effects using the EVAL14 [21] software. Final cell parameters were obtained after refining the whole data set. The data were then reintegrated and corrected for absorption using the SADABS [22] program and finally merged with the software XPREP [23]. Crystal and data collection details are given in Table S1. The structure was solved by intrinsic phasing and refined by full-matrix least square methods with, respectively, the SHELXT-2016 and SHELXL-2019/3 programs [24] implemented in Olex2 software [25]. C, F, N, O, P, and Ru atoms were refined with anisotropic thermal parameters. H atoms were set geometrically, riding on the carrier atoms, with isotropic thermal parameters. CCDC- 2359054-2359055 contain the crystallographic data for [Ru(**L**)_2_](PF_6_)_2_ and [Rutpy(**L**)](PF_6_)_2_, respectively; the data can be obtained free of charge via https://www.ccdc.cam.ac.uk/structures/? (accessed on 18 July 2024).

### 3.4. Synthesis of Compounds

#### 3.4.1. Synthesis of Ligand **L**

Ligand **L**: To a solution of 4,5-dibromo-2,7-di-*tert*-butylacridine (900 mg, 2 mmol) and 1*H*-pyrazole (288 mg, 4.2 mmol) in dry dimethylformamide (DMF) (8 mL), *L*-proline (464 mg, 4.04 mmol), anhydrous K_2_CO_3_ (1.145 g, 8.3 mmol), and copper(I) iodide (380 mg, 2 mmol) were added. The solution mixture was heated at 120 °C for 20 h under argon. After cooling to room temperature, 40 mL of distilled water was added. The organic phase was extracted with ethyl acetate (3 × 60 mL). The organic extracts were combined and dried on sodium sulfate and all of the volatile substances were removed under vacuum. The obtained dark brown crude mixture was purified by flash column chromatography (SiO_2_; EtOAc/Pentane 10%, Rf = 0.31), and the desired bright yellowish pure compound was isolated (335 mg, 40% yield). ^1^H-NMR (400 MHz; acetone-*d*_6_): δ 9.21 (s, 1H; H_9a_), 8.81 (d, *J* = 2.0 Hz, 2H; H_3pz_), 8.46 (d, *J* = 1.5 Hz, 2H; H_3a_), 8.11 (d, *J* = 1.5 Hz, 2H; H), 7.80 (d, *J* = 2.0 Hz, 2H; H_5pz_), 6.53 (t, *J* = 1.8 Hz, 2H; H_4pz_), 1.53 (s, 18H; H_t-Bu_). ^13^C-NMR (101 MHz, CDCl_3_): δ 149.0, 140.5, 139.9, 136.8, 136.3, 134.0, 127.6, 124.0, 121.4, 106.4, 35.4, 30.9. ESI-MS: *m*/*z* = 424.20 ([M+H]^+^), calculated for C_27_H_29_N_5_H^+^ = 424.25; *m*/*z* = 446.20 ([M+Na]^+^), calculated for C_27_H_29_N_5_Na^+^ = 446.23.

#### 3.4.2. Synthesis of [Ru(L)_2_](PF_6_)_2_

[RuCl_2_(dmso)_4_] (30 mg, 0.062 mmol) and ligand **L** (58 mg, 0.137 mmol) were dissolved in dry ethanol (2 mL). The reaction mixture was refluxed overnight. After cooling to room temperature, a precipitate of ruthenium(II) complex (in the form of hexafluorophosphate salt) was obtained by the addition of an excess of the saturated aqueous solution of KPF_6_. The resulting solid was purified by column chromatography (SiO_2_; acetone/H_2_O/KNO_3(sat)_ 100/5/0.5 *v*/*v*/*v*, Rf = 0.3), affording the desired complex as a light blue powder (60 mg, 35% yield). ^1^H-NMR (500 MHz; acetone-*d*_6_): δ 9.08 (s, 2H; H_9a_), 9.00 (d, *J* = 2.1 Hz, 2H; H_3pz_), 8.99 (d, *J* = 2.9 Hz, 2H; H_5pz_), 8.49 (d, *J* = 1.7 Hz, 2H; H_3a_), 8.41 (d, *J* = 2.9 Hz, 2H; H5′_pz_), 8.27 (d, *J* = 2.1 Hz, 2H; H_3′pz_), 8.21 (d, *J* = 1.5, Hz, 2H; H_8a_), 7.89 (d, *J* = 1.5 Hz, 2H; H_1a_), 7.62 (d, *J* = 1.7 Hz, 2H; H_6a_), 7.19 (t, *J* = 2.65 Hz, 2H; H_4pz_), 6.77 (t, *J* = 2.65 Hz, 2H; H_4′pz_), 1.60 (s, 18H), 1.53 (s, 18H). ^13^C-NMR (101 MHz, CD_3_CN): δ 152.1, 150.3, 148.3, 147.5, 147.4, 146.4, 136.5, 136.4, 134.8, 133.1, 130.2, 126.9, 126.8, 125.4, 123.5, 122.7, 122.2, 111.9, 111.2, 36.3, 36.1, 31.1, 30.8. ESI-MS: *m*/*z* = 474.20 ([Ru(**L**)_2_]^2+^), calculated for C_54_H_58_N_10_Ru^2+^ = 474.19; *m*/*z* = 1093.30 ([Ru(**L**)_2_](PF_6_)^+^), calculated for C_54_H_58_F_6_N_10_PRu^+^ = 1093.35.

#### 3.4.3. Synthesis of [Ru(tpy)L](PF_6_)_2_

[RuCl_3_(tpy)] (66 mg, 0.15 mmol) and ligand **L** (64 mg, 0.15 mmol) were completely dissolved in 10 mL of an ethanol/water mixture (7/3 *v*/*v*). Triethylamine (0.2 mL, 1.44 mmol) was then added to the solution and the mixture was allowed to reflux for 4 h. After cooling to room temperature, a dark precipitate of ruthenium(II) complex (in the form of hexafluorophosphate salt) was obtained by the addition of an excess of the saturated aqueous solution of KPF_6_. Column chromatography purification (SiO_2_; acetone/H_2_O/KNO_3(sat)_ 100/8/0.8 *v*/*v*/*v*, Rf = 0.25) afforded the dark blue Ru(II) complex (46 mg, 29% yield). ^1^H-NMR (500 MHz; acetone-*d*_6_): δ 9.68 (s, 1H; H_9a_), 8.93 (d, *J* = 8.1 Hz, 2H), 8.75 (dt, *J* = 8.1, 0.8 Hz, 2H), 8.58 (d, *J* = 2.1 Hz, 2H; H_3a_), 8.52 (t, *J* = 8.1 Hz, 1H), 8.45 (dd, *J* = 3.0, 0.8 Hz, 2H; H_1a_), 8.44 (d, *J* = 2.0 Hz, 2H; H_5pz_), 8.14 (td, *J* = 7.9, 1.5 Hz, 2H), 8.06 (ddd, *J* = 5.6, 1.5, 0.6 Hz, 2H), 7.37 (ddd, *J* = 7.9, 5.6, 1.3 Hz, 2H), 6.95 (dd, *J* = 2.4, 0.6 Hz, 2H; H_3pz_), 6.48 (td, *J* = 2.7, 0.5 Hz, 2H; H_4pz_), 1.57 (s, 18H; H_t-Bu_). ^13^C-NMR (125 MHz; acetone-*d*_6_): δ 159.3, 159.1, 153.8, 151.0, 143.1, 141.2, 140.3, 139.4, 138.0, 136.6, 131.5, 130.0, 129.9, 128.0, 125.6, 124.9, 124.0, 110.9, 42.7, 35.7. ESI-MS: *m*/*z* = 379.07 ([Ru(tpy)**L**]^2+^), calculated for C_42_H_40_N_8_Ru^2+^ = 379.12.

## 4. Conclusions

A novel acridine-based tridentate ligand comprising two pyrazole moieties was synthesized along with two ruthenium(II) complexes derived from it: one homoleptic bearing two acridine ligands and the other one, heteroleptic, in which ligand **L** was combined with an ancillary terpyridine ligand. Their photophysical, electrochemical, and structural properties were determined, and this set of experimental evidence, along with an NMR study, showed that the ligand can coordinate in two different fashions. It displays a *fac* coordination mode in the homoleptic complex [Ru(**L**)_2_]^2+^; however, in the heteroleptic complex [Ru(tpy)(**L**)]^2+^, the coordination of the tpy ligand imposes a *mer* coordination of **L**. Despite favorable geometrical features and adequate electronic levels, both complexes were non-luminescent.

## Figures and Tables

**Table 1:** Bond lengths and angles in the coordination sphere.

Bond Angles	[Ru(L)_2_](PF_6_)_2_	[Ru(tpy)L](PF_6_)_2_
N1-Ru-N2	85.17(16)	89.03(16) ^1^
N1-Ru-N3	93.99(17)	178.95(18) ^1^
N1-Ru-N4	175.57(18)	89.02(17)
N1-Ru-N5	91.14(16)	91.14(17)
N1-Ru-N6	89.48(17)	90.72(17)
N2-Ru-N3	85.96(18)	90.57(17) ^1^
N2-Ru-N4	91.73(19)	100.22(17)
N2-Ru-N5	92.97(17)	179.8(2)
N2-Ru-N6	174.20(18)	100.01(17)
N3-Ru-N4	88.94(19)	92.01(17)
N3-Ru-N5	174.65(18)	89.27(18)
N3-Ru-N6	96.66(18)	88.39(17)
N4-Ru-N5	85.86(18)	79.8(2) ^2^
N4-Ru-N6	93.5(2)	159.76(19) ^2^
N5-Ru-N6	84.89(17)	80.0(2) ^2^
**Bond Lengths**		
Ru-N1	2.044(4)	2.034(5) ^1^
Ru-N2	2.047(5)	2.101(5) ^1^
Ru-N3	2.047(5)	2.041(5) ^1^
Ru-N4	2.036(5)	2.074(5) ^2^
Ru-N5	2.054(4)	1.953(5) ^2^
Ru-N6	2.047(5)	2.076(5) ^2^

^1^ Angles and bond lengths within ligand **L**. ^2^ Angles and bond lengths within the tpy ligand. All other values are inter-ligand angles.

**Table 2:** UV–visible absorption data of complexes [Ru(**L**)_2_]^2+^ and [Ru(tpy)**L**]^2+^ in acetonitrile solution.

Complex	λ_abs_, nm (ε, M^−1^∙cm^−1^)
[Ru(**L**)_2_]^2+^	228 (50,000), 265 (53,100), 307 (13,300), 404 (2500), 448 (2100), 540 (1600), 747 (2000)
[Ru(tpy)**L**]^2+^	229 (45,000), 272 (53,000), 316 (28,500), 405 (3300), 427 (3500), 514 (6900), 580 (6000)

**Table 3:** Redox potentials of the studied complexes in acetonitrile along with those of [Ru(tpy)_2_]^2+^ for comparison. *E*_1/2_ (V) = (*E*_pa_ + *E*_pc_)/2; (Δ*E_p_* (mV) = *E*_pc_ − *E*_pa_) vs. Ag^+^(0.01 M)/Ag. The concentration is 10^−3^ M; sweep rate = 100 mV×s^−1^.

Complex	E_1/2_ Reduction (ΔE_p_, mV)		E_1/2_ Oxidation (ΔE_p_, mV)
	L	tpy	Ru
[Ru(tpy)_2_]^2+^		−1.83 (70); −1.57 (60)	0.97 (80)
[Ru(**L**)_2_]^2+^	−1.51 *; −1.06 *		0.64 (65)
[Ru(tpy)**L**]^2+^	−2.30 *; −1.22 *	−1.74 (80)	0.82 (60)

* Irreversible processes.

**Table 4:** Estimated HOMO-LUMO energy gap from electronic absorption spectra and cyclic voltammetry data for complexes [Ru(**L**)_2_]^2+^ and [Ru(tpy)**L**]^2+^.

Complex	UV-vis		CV		
	λ, nm	∆*E* *_HOMO-LUMO_, eV	*E*_pa_, V	*E*_pc_, V	∆E_HOMO-LUMO_, eV
[Ru(**L**)_2_]^2+^	747	1.66	0.64	−1.06	1.70
[Ru(tpy)**L**]^2+^	580	2.14	0.82	−1.22	2.04

* Based on the lowest energy absorption band using the Planck formula.

## Data Availability

The original contributions presented in the study are included in the article/Supplementary Materials, further inquiries can be directed to the corresponding authors.

## Supplementary Materials

The following supporting information can be downloaded at: https://www.mdpi.com/article/10.3390/molecules29153468/s1, Table S1: Crystal data and structure refinement; Figure S1. NMR spectrum of ligand **L**; Figure S2. COSY-NMR spectrum of [Ru(**L**)_2_](PF_6_)_2_; Figure S3. NOESY-NMR spectrum of [Ru(**L**)_2_](PF_6_)_2_; Figure S4. COSY-NMR spectrum of [Ru(tpy)**L**](PF_6_)_2_; Figure S5. NOESY-NMR spectrum of [Ru(tpy)**L**](PF_6_)_2_; Figure S6: Cyclic voltammogram of complex [Ru(**L**)_2_](PF_6_)_2_; Figure S7: Cyclic voltammogram of complex [Rutpy(**L**)](PF_6_)_2_.
