# Supplementary material for: Tuning the Coordination Environment of Ru(II) Complexes with a Tailored Acridine Ligand
Source: Molecules. 2024 Jul 24;29(15):3468. doi: 10.3390/molecules29153468 (PMC11313782; doi:10.3390/molecules29153468)
Supplement: Supplementary file 1 [file molecules-29-03468-s001.zip › molecules-3069323-supplementary.pdf]

Supplementary Information

Tuning the Coordination Environment of Ru(II) Complexes with a Tailored Acridine Ligand

Ali Awada, Pierre-Henri Lanoë, Christian Philouze, Frédérique Loiseau* and Damien Jouvenot*

1. X-ray Diffraction

*Table S1: Crystal data and structure refinement for [Ru(**L**)$_2$](PF$_6$)$_2$ and [Ru(tpy)**L**](PF$_6$)$_2$.........2*

2. NMR Spectra

2.1. Ligand

*Figure S1. NMR spectrum of ligand **L** in acetone-d6 at 500 MHz...3*

2.2. [Ru(**L**)$_2$](PF$_6$)$_2$

*Figure S2. COSY-NMR spectrum of [Ru(**L**)$_2$](PF$_6$)$_2$ in acetone-d6 at 500 MHz............................4*

*Figure S3. NOESY-NMR spectrum of [Ru(**L**)$_2$](PF$_6$)$_2$ in acetone-d6 at 500 MHz.........................4*

2.3. [Ru(tpy)**L**](PF$_6$)$_2$

*Figure S4. COSY-NMR spectrum of [Ru(tpy)**L**](PF$_6$)$_2$ in acetone-d6 at 500 MHz........................5*

*Figure S5. NOESY-NMR spectrum of [Ru(tpy)**L**](PF$_6$)$_2$ in acetone-d6 at 500 MHz.....................5*

3. Cyclic voltammetry

3.1. [Ru(**L**)$_2$](PF$_6$)$_2$

*Figure S6 : Cyclic voltammogram of complex [Ru(**L**)$_2$](PF$_6$)$_2$ in a 10^{-3} M deaerated acetonitrile solution + 0.1 M [BuN]$_4$PF$_6$ (TBAPF$_6$), at a scan rate of 100 mV.s^{-1} using vitreous carbon electrode (5 mm diameter) as working electrode, E_p(V) vs AgNO$_3$ (0.01 M)/Ag...........................6*

3.2. [Ru(tpy)**L**](PF$_6$)$_2$

*Figure S7 : Cyclic voltammogram of complex [Rutpy(**L**)](PF$_6$)$_2$ in a 10^{-3} M deaerated acetonitrile solution + 0.1 M [BuN]$_4$PF$_6$ (TBAPF$_6$), at a scan rate of 100 mV s^{-1} using vitreous carbon electrode (5 mm diameter) as working electrode, E_p(V) vs AgNO$_3$ (0.01 M)/Ag.........................7*

1. X-ray Diffraction

*Table S1: Crystal data and structure refinement for [Ru(**L**)$_2$](PF$_6$)$_2$ and [Ru(tpy)**L**](PF$_6$)$_2$.*

Compound	[Ru(tpy)**L**](PF$_6$)$_2$	[Ru(**L**)$_2$](PF$_6$)$_2$
Formula	$C_{42}H_{40}N_8Ru$. 2(PF$_6$), (C_3H_60)	$C_{54}H_{58}N_{10}Ru$. 2(PF$_6$), (C_3H_60)
F_w [g.mol^{-1}]	1105,91	1296.19
T [K]	200	200
Morphology	needle	plate
Color	violet	black
Crystal size mm	0.12 x 0.22 x 0.50	0.16 x 0.36 x 0.58
Crystal system	Monoclinic	Orthorhombic
Space group	*C2/c*	*Pbca*
a [Å]	26.357(5)	16.851(3)
b [Å]	9.3026(19)	16.362(3)
c [Å]	40.918(8)	43.245(9)
α [°]	90	90
β [°]	108.24(3)	90
γ [°]	90	90
Unit-cell volume [Å^3]	9528(4)	11923(4)
Z	8	8
D_x [g·cm^{-3}]	1.542	1.444
μ [mm^{-1}]	0.488	0.403
F(000)	4496	5328
Radiation [Å]	MoKα (λ = 0.71073)	MoKα (λ = 0.71073)
Θ range for data collection/°	2.336 to 25.000	1.974 to 25.000
Index ranges	−31 ≤ h ≤ 31, −11 ≤ k ≤ 11, −48 ≤ l ≤ 47	−20 ≤ h ≤ 20, −19≤ k ≤ 19, −51 ≤ l ≤ 51
Total reflections	51456	97177
Unique reflections	8239	10407
Used reflections (I>2σ(I))	6352	8029
Refined parameters	837	965
Rint.	0.0855	0.0699
R1	0.0680	0.0688
R(w)[a]	0.1477	0.1608
Goodness of fit *S*	1.149	1.271
$\Delta\rho_{min}/\Delta\rho_{max}$ (e·Å^{-3})	-0.755/0.626	-0.599/0.535

[a] Refinement based on F^2 where $w = 1/[\sigma^2(Fo^2)+(0.0546P)^2+62.3962P]$ where $P=(Fo^2+2Fc^2)/3$ for [Rutpy(**L**)](PF$_6$)$_2$
$w = 1/[\sigma^2(Fo^2)+(0.0443P)^2+38.1714P]$ where $P=(Fo^2+2Fc^2)/3$ for [Ru(**L**)$_2$](PF$_6$)$_2$

2. NMR Spectra

2.1. Ligand

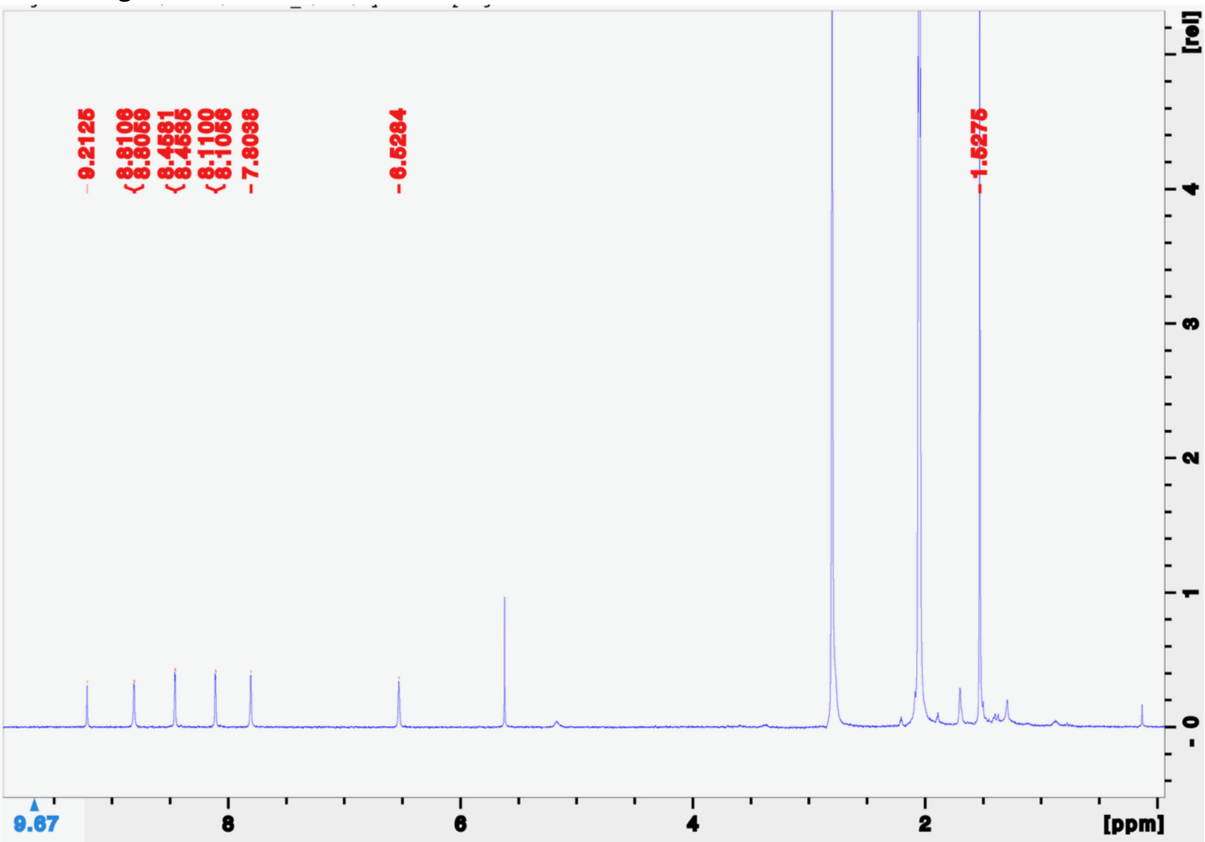

*Figure S1. NMR spectrum of ligand **L** in acetone-d_6 at 500 MHz.*

2.2. [Ru(**L**)$_2$](PF$_6$)$_2$

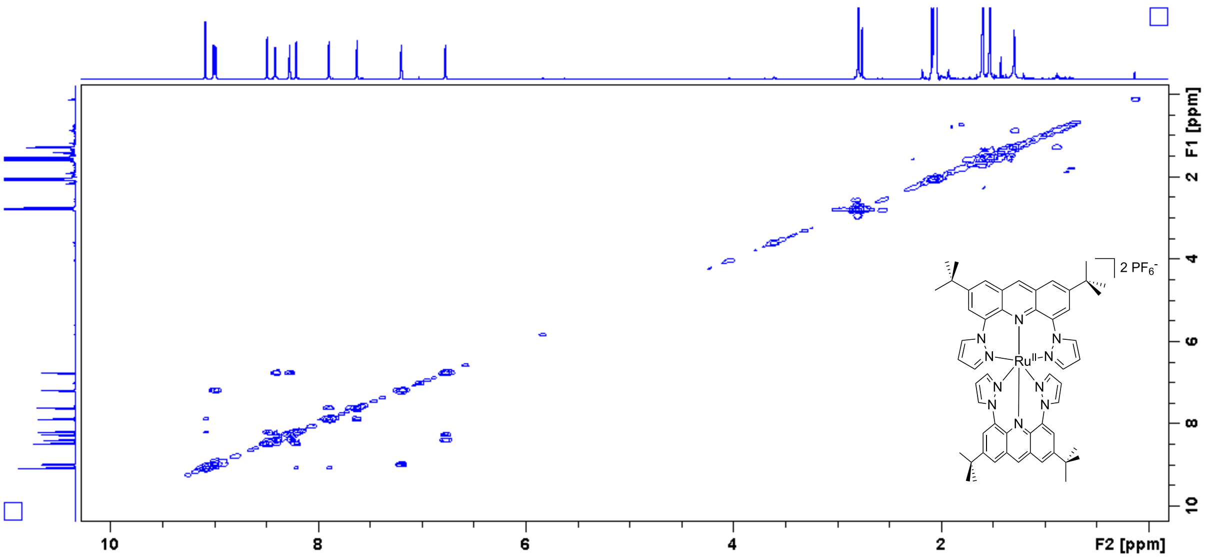

*Figure S2. COSY-NMR spectrum of [Ru(**L**)$_2$](PF$_6$)$_2$ in acetone-d$_6$ at 500 MHz.*

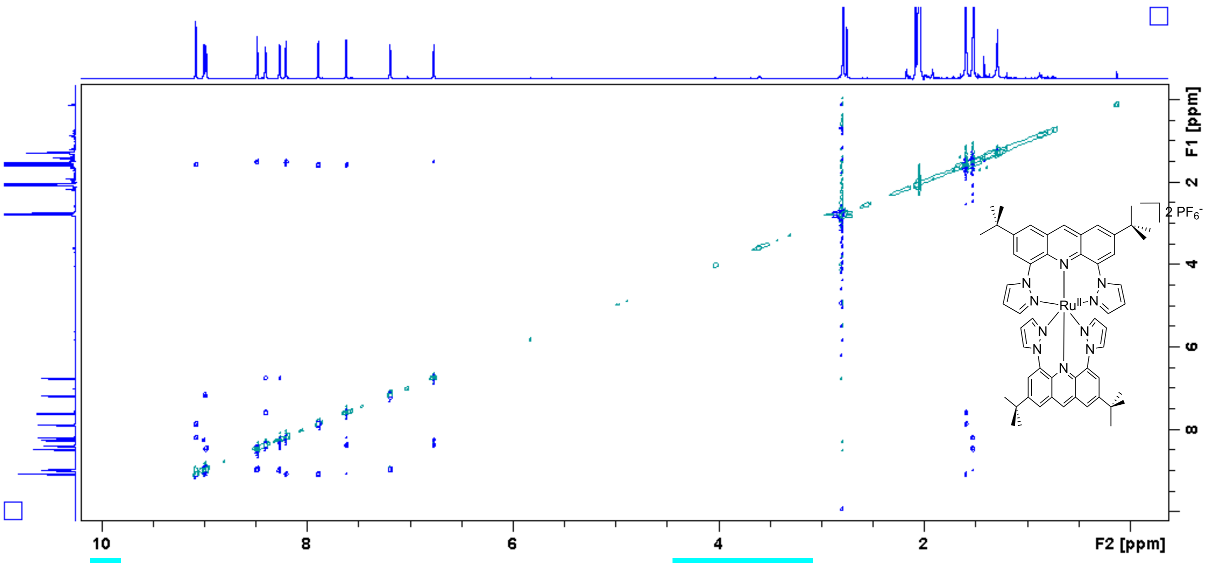

*Figure S3. NOESY-NMR spectrum of [Ru(**L**)$_2$](PF$_6$)$_2$ in acetone-d$_6$ at 500 MHz.*

2.3. [Ru(tpy)**L**]$(PF_6)_2$

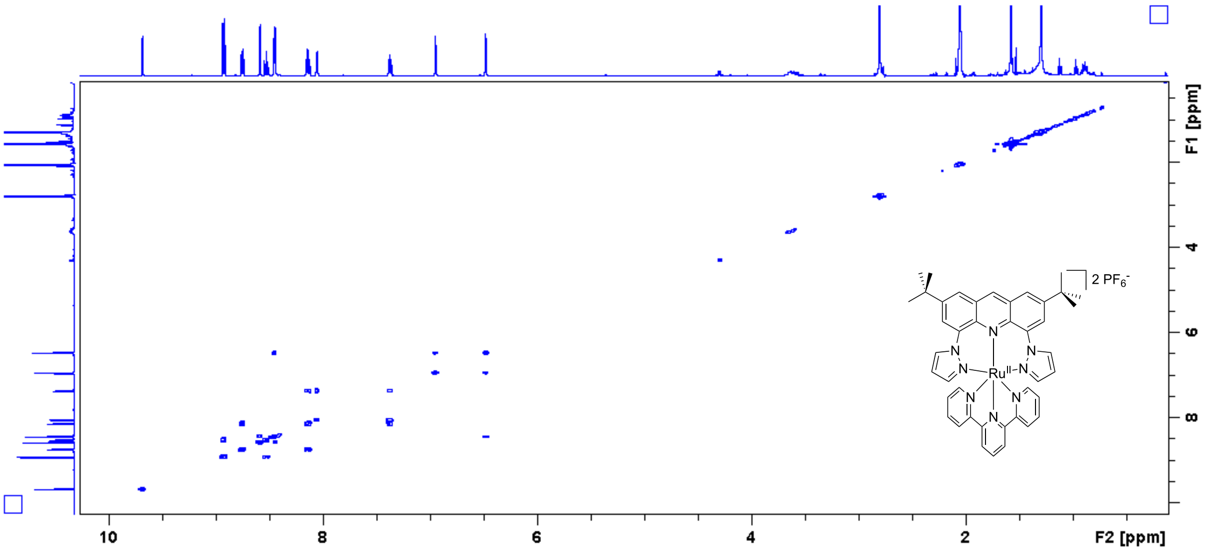

*Figure S4. COSY-NMR spectrum of [Ru(tpy)**L**]$(PF_6)_2$ in acetone-d_6 at 500 MHz.*

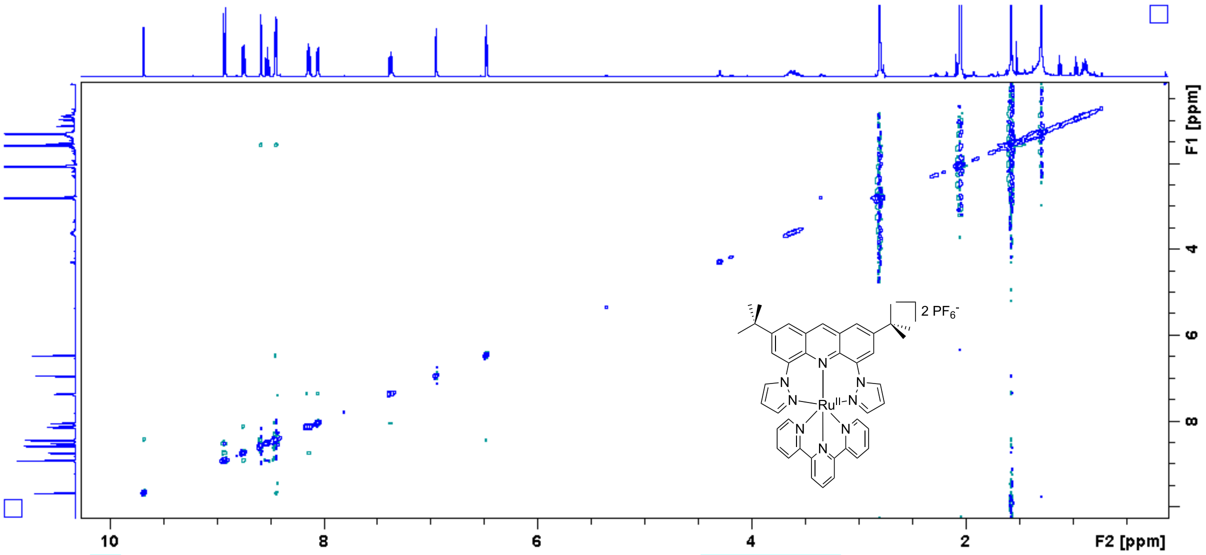

*Figure S5. NOESY-NMR spectrum of [Ru(tpy)**L**]$(PF_6)_2$ in acetone-d_6 at 500 MHz.*

3. Cyclic voltammetry

3.1. $[Ru(\mathbf{L})_2](PF_6)_2$

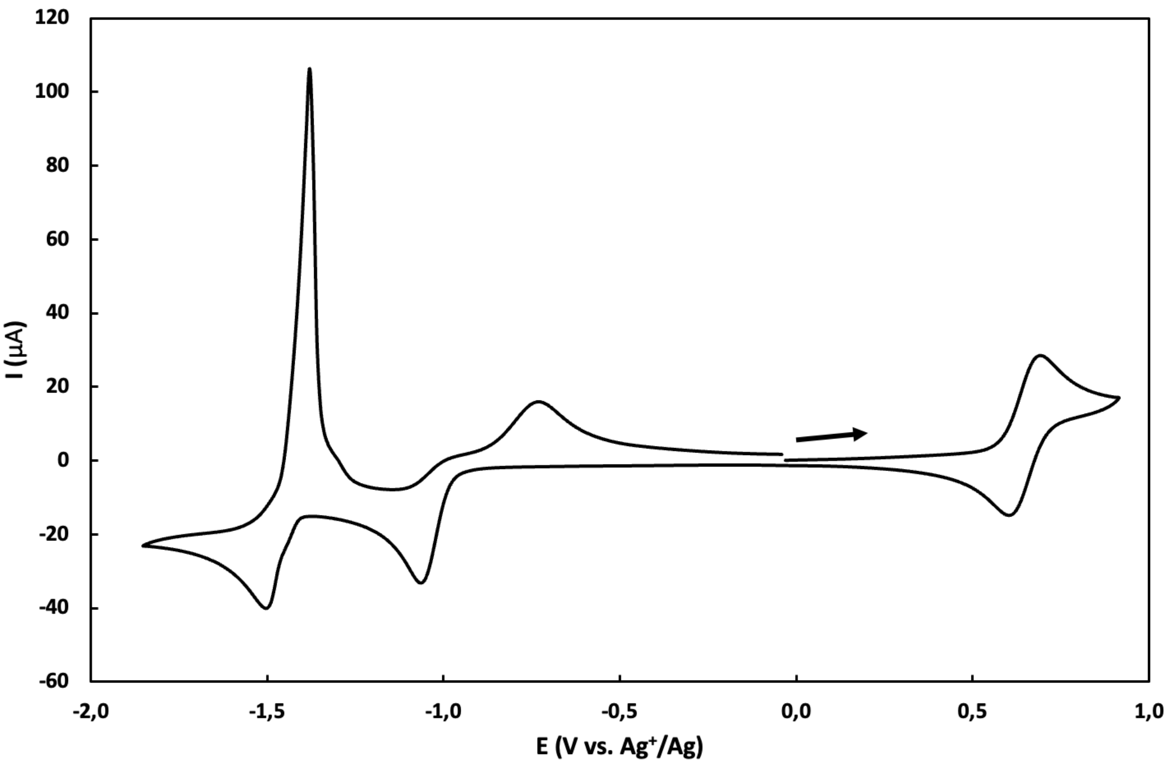

Figure S6 : Cyclic voltammogram of complex $[Ru(\mathbf{L})_2](PF_6)_2$ in a 10^{-3} M deaerated acetonitrile solution + 0.1 M $[n\text{-}Bu_4N]PF_6$ ($TBAPF_6$), at a scan rate of 100 mV s^{-1} using vitreous carbon electrode (5 mm diameter) as working electrode, E_p(V) vs $AgNO_3$ (0.01 M)/Ag. Reversible oxidation process: $I_a/I_c = 1$.

3.2. [Ru(tpy)**L**](PF_6)$_2$

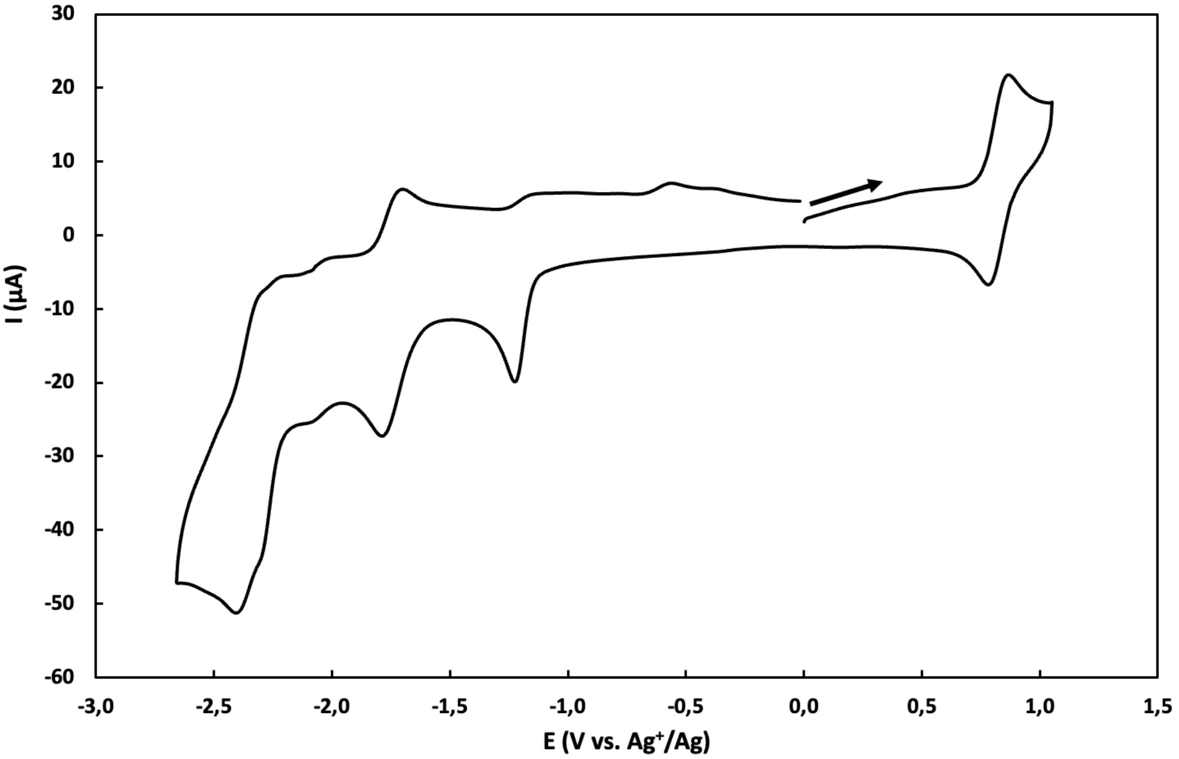

*Figure S7 : Cyclic voltammogram of complex [Rutpy(**L**)](PF_6)$_2$ in a 10^{-3} M deaerated acetonitrile solution + 0.1 M [n-Bu_4N]PF_6 (TBAPF_6), at a scan rate of 100 mV s^{-1} using vitreous carbon electrode (5 mm diameter) as working electrode, E_p(V) vs $AgNO_3$ (0.01 M)/Ag. Reversible oxidation process: I_a/I_c = 1.2.*
